# Risk factors for late death of patients with abdominal trauma after damage control laparotomy for hemostasis

**DOI:** 10.1186/1749-7922-9-1

**Published:** 2014-01-04

**Authors:** Li-Min Liao, Chih-Yuan Fu, Shang-Yu Wang, Chien-Hung Liao, Shih-Ching Kang, Chun-Hsiang Ouyang, I-Ming Kuo, Shang-Ju Yang, Yu-Pao Hsu, Chun-Nan Yeh, Shao-Wei Chen

**Affiliations:** 1 Department of Trauma and Emergency Surgery, Chang Gung Memorial Hospital, 5 Fu-Hsing Street, Taoyuan, Kwei Shan Township, Taiwan; 2 Graduate Institute of Clinical Medical Sciences, Chang Gung University, 259 Wen-Hwa 1st Road, Taoyuan, Kwei Shan Township, Taiwan; 3 Department of General Surgery, Chang Gung Memorial Hospital, 5 Fu-Hsing Street, Taoyuan, Kwei Shan Township, Taiwan; 4 Department of Cardiovascular and Thoracic Surgery, Chang Gung Memorial Hospital, 5 Fu-Hsing Street, Taoyuan, Kwei Shan Township, Taiwan

**Keywords:** Abdominal trauma, Damage control laparotomy, Damage control surgery

## Abstract

**Introduction:**

In this study, we explored the possible causes of death and risk factors in patients who overcame the initial critical circumstance when undergoing a damage control laparotomy for abdominal trauma and succumbed later to their clinical course.

**Methods:**

This was a retrospective study. We selected patients who fulfilled our study criteria from 2002 to 2012. The medical and surgical data of these patients were then reviewed. Fifty patients (survival vs. late death, 39 vs. 11) were enrolled for further analysis.

**Results:**

In a univariable analysis, most of the significant factors were noted in the initial emergency department (ED) stage and early intensive care unit (ICU) stage, while an analysis of perioperative factors revealed a minimal impact on survival. Initial hypoperfusion (pH, BE, and GCS level) and initial poor physiological conditions (body temperature, RTS, and CPCR at ED) may contribute to the patient’s final outcome. An analysis and summary of the causes of death were also performed.

**Conclusions:**

According to our study, the risk factors for late death in patients undergoing DCL may include both the initial trauma-related status and clinical conditions after DCL. In our series, the cause of death for patients with late mortality included the initial brain insult and later infectious complications.

## Introduction

Damage control laparotomy (DCL) has been adopted as a life-saving and temporary procedure for dying patients who have sustained a major trauma and undergone other abdominal emergency [1-4]. DCL is performed with an initial laparotomy with gauze packing for hemorrhage control, vascular pedicle ligation, or contamination control. After the initial emergent management, patients are sent to the intensive care unit (ICU) to correct unfavorable factors, such as hypothermia, coagulopathy, acidosis, and electrolyte imbalances. Within 48 to 72 hours after the first laparotomy, a second laparotomy is usually performed for definitive treatment. DCL was first applied in patients with hepatic injuries during the early 20th century, and this technique was further refined decades later [1]. Currently, DCL is widely used in the emergency setting for patients with uncontrolled intra-abdominal bleeding or severely contaminated intestinal or urological trauma. With recent advances in ICU management, DCL is usually followed by organized and protocolized treatment plans, bridging the initial damage control procedure to definite treatment [5].

DCL provides critically ill patients with the best chance of survival, expands the interval for other life-saving interventions, and prepares patients for a secondary laparotomy. Between the first damage control procedure and the secondary laparotomy, ICU physicians always make their best effort to develop a thorough treatment plan, from maintaining the patient with good oxygenation to the sophisticated tuning of resuscitation details [6]. In addition, adjuvant hemostatic procedures, such as trans-arterial embolization (TAE) [7], are sometimes necessary for better hemostatic effect. Even with advanced ICU management and successful hemostasis, however, some of those patients still succumb later to their complicated clinical course. In this study, we will explore the possible causes of death and risk factors in patients who survived the initial critical circumstance but succumbed to the later clinical course.

## Methods and materials

### Clinical setting

Chang Gung Memorial Hospital (CGMH) is a level I trauma center in northern Taiwan. From May 2008 to June 2012, 1203 patients sustained abdominal trauma, and 336 patients underwent surgery (either a laparotomy or a laparoscopic procedure). At CGMH, we not only have a 24-hour specialized trauma team but also have standard protocols for all different types of major trauma over 10 years. In addition, emergent TAE is widely used in our institute and has been available at any hour for the past decade. For patients with solid organ injury (including hepatic, renal, and splenic injuries), approximately 90% of non-operative management was conducted with a low failure rate (< 2%). For patients with intra-abdominal bleeding, we only performed laparotomy for refractory hemorrhagic shock, multiple bleeding sites with difficult TAE approaching, and either a complete failure or temporary benefit of TAE.

### Inclusion criteria

In this study, we excluded patients aged less than 18 and over 65, patients who arrived at the emergency department (ED) 6 hours after the traumatic incident, pregnant patients, patients with end-stage renal disease, and patients with congestive heart failure. In addition, we also excluded patients who underwent DCL after ICU admission or later during their hospital stay. Only patients who suffered from blunt or penetrating abdominal trauma and were later sent to operation room (OR) directly from the ED were enrolled for further analysis. We defined late death as patients who died 48 hours or later after DCL with successful hemostasis.

### Study design

**Figure 1:**
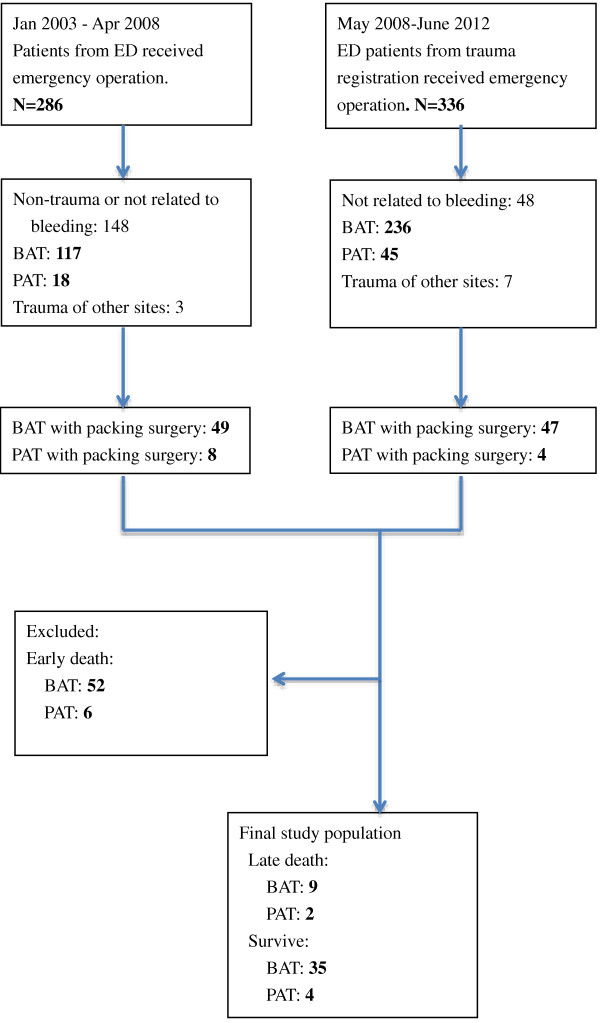
Flowchart for the selection of the studied patients.

This was a retrospective study and was approved by the local institutional review board of CGMH. The Trauma Registration System of CGMH was started from May 2008. We selected patients fulfilling our criteria in the database from May 2008 to June 2012. These patients all suffered from abdominal trauma and damage control laparotomies with gauze-packing. For the pre-registration period, from January 2002 to April 2008, we accessed the OR information system to retrieve the list of patients who underwent emergent laparotomy and fulfilled our study criteria. The medical and surgical data of these patients were then reviewed. Fifty patients (survival vs. late death, 39 vs. 11) enrolled for further analysis (Figure 1).

Demographic data, clinical profile, laboratory data, and radiologic reports were all evaluated by two surgical residents and two attending surgeons. Patients’ identification, mechanism of trauma, initial status in the ED, initial laboratory data, transfusion volume, status when leaving the ED, injury severity score (ISS), revised trauma score (RTS), surgical conditions, significant ICU interventions, diagnosis, and outcome were all extracted for further analysis. All patients were categorized into 2 groups: the survival group (n = 39) and the late death group (n = 11). Comparisons between these 2 groups were performed first, and significant factors from the univariable analysis were further analyzed in a multivariable analysis.

### Statistical analysis

This analysis used the SPSS statistical software package, version 20.0. The Mann–Whitney U test was used to evaluate numerical variables, and either the χ2 test or Fisher’s exact test was used for nominal data. Logistic regression was used for the multivariable analysis. Significance was defined as *p* < 0.05.

## Results

### Demographic data and clinical conditions upon ED arrival

The demographic data and initial status when the patients arrived at the ED were analyzed and are summarized in Table 1. The initial body temperature, Glasgow Coma Scale (GCS) less than 8, RTS, initial cardiopulmonary and cerebral resuscitation (CPCR), pH, and base excess (BE) were all noted with statistical significance. In addition, the total numbers of laparotomies were similar between the two groups.

**Table 1:** Demographic data and initial ED condition of patients

	**Survival (mean±SD, n-=39)**	**Late death (mean±SD, n=11)**	***p***
Gender (M/F)	30/9	10/1	n.s.
Age (y/o)	33.3 ± 4.98	42.8 ± 13.0	n.s.
Transfer (Y/N)	27/12	7/4	n.s.
Time from accident (min)	162 ± 46.4	136 ± 53.1	n.s.
Blunt injury (Y/N)	35/4	9/2	n.s.
BT (°C)	36.0 ± 0.41	35.0 ± 0.83	0.017
HR (/min)	111.3 ± 8.52	100.5 ± 25.5	n.s.
RR (/min)	21.8 ± 2.44	21.1 ± 4.28	n.s.
SBP (mmHg)	90.1 ± 12.0	76.8 ± 28.2	n.s.
DBP (mmHg)	57.8 ± 8.68	43.2 ± 20.9	n.s.
GCS < =8 (Y/N)	7/32	6/5	0.023
RTS	6.31 ± 0.45	4.89 ± 1.24	0.032
CPCR at ED (Y/N)	0/39	3/8	0.008
Hb (g/dl)	9.98 ± 0.83	9.08 ± 1.90	n.s.
pH	7.29 ± 0.03	7.09 ± 0.13	0.004
HCO_3_ (meq/l)	18.6 ± 1.42	16.6 ± 0.13	n.s.
BE (mmol/l)	−7.96 ± 1.65	−13.2 ± 4.16	0.026
INR	1.72 ± 0.22	2.21 ± 0.68	n.s.
ISS	30.4 ± 4.70	32.1 ± 9.04	n.s.
Total laparotomy times	2.28 ± 1.45	2.27 ± 0.93	n.s.

Statistical significant was defined as p < 0.05. *SD*, standard deviation; *BT*, Body temperature; *HR*, Heart rate; *RR*, Respiratory rate; *SBP*, Systolic blood pressure; *DBP*, Diastolic blood pressure; *GCS*, Glasgow Coma Scale; *RTS*, Revised trauma score; *CPCR*, Cardiopulmonary cerebral resuscitation; *Hb*, Hemoglobin; *BE*, Base excess; *INR*, International normalized ratio, for prothrombin time; *ISS*, Injury severity score.

### Perioperative conditions

Preoperative and intra-operative conditions are summarized in Table 2. Except the preoperative GCS, the 2 study groups showed no differences among the analyzed factors. Although not statistically significant, the major bleeding site seemed to be the liver (36.0% in the survival group vs. 45.5% in the late death group). In addition, the percentage of patients with late death who underwent associate procedures for hemostasis (thoracotomy or external fixation for pelvic fracture) was greater than that of survival group (36.5% vs. 8.3%, respectively).

**Table 2:** Preoperative status of patients

	**Survival (mean±SD, n-=39)**	**Late death (mean±SD, n=11)**	***p***
Time to OR (min)	124 ± 35.4	128 ± 37.5	n.s.
RR (/min)	22.2 ± 1.64	21.7 ± 3.10	n.s.
HR (/min)	119 ± 4.16	116 ± 7.70	n.s.
SBP (mmHg)	100 ± 11.7	101 ± 10.6	n.s.
DBP (mmHg)	58.7 ± 6.78	56.6 ± 6.18	n.s.
GCS < =8 (Y/N)	12/27	9/2	0.040
Major bleeding site			
Liver	14	5	n.s.
Spleen	8	4	
Pelvis	2	0	
Mesentery	4	1	
Kidney	2	0	
Multiple	8	1	
Others	1	0	
Perioperative TAE (Y/N)	12/27	4/7	n.s.
Associated procedure(s) for hemostasis	3/36	3/8	n.s.

Statistical significant was defined as p < 0.05. *SD*, Standard deviation; *OR*, Operation room; *HR*, Heart rate; *RR*, Respiratory rate; *SBP*, Systolic blood pressure; *DBP*, Diastolic blood pressure; *GCS*, Glasgow Coma Scale; *TAE*, Trans-arterial embolization.

### ICU parameters and interventions

The analysis of the post-DCL ICU parameters is summarized in Table 3. The most analyzed factors were the best data recorded within 48 hours after DCL. Hemodialysis and extracorporeal membrane oxygenation (ECMO) use in our study refers to the applications of those modalities at any time during the ICU course, while the accumulated blood transfusion refers to volume of packed red blood cells and whole blood that was administered in the b agent, white cell count (WBC), lowest FiO2 use, INR, use of hemodialysis or ECMO, and accumulated blood transfusion volume were all noted with statistical significance.

**Table 3:** Early clinical parameters and organ support system application in ICU

	**Survival (mean ± SD, n = 39)**	**Late death (mean ± SD, n = 11)**	***p***
APACHI II	14.8 ± 1.33	22.4 ± 3.19	0.000
Best GCS > = 8 (Y/N)	37/2	6/5	0.004
Inotropic agent use (Y/N)	7/32	11/0	0.000
Best PaO_2_ (mmHg)	68.8 ± 6.77	76.4 ± 9.33	n.s.
Lowest FiO_2_ (%)	240 ± 42.5	251 ± 112	n.s.
WBC (10^3^/dl)	13.3k ± 5.66k	7.29k ± 5.57k	0.020
Hb (g/dl)	11.4 ± 0.32	11.0 ± 1.63	n.s.
PLT (10^3^/dl)	88.6k ± 17.7k	94.4k ± 36.8k	n.s.
INR	1.47 ± 0.89	1.81 ± 0.33	0.016
Na (meq/l)	143 ± 7.41	151 ± 2.89	n.s.
K (meq/l)	3.76 ± 0.29	3.83 ± 0.53	n.s.
Cr (mg/dl)	1.27 ± 0.44	1.10 ± 0.27	n.s.
Total bilirubin (mg/dl)	1.44 ± 0.46	1.27 ± 0.47	n.s.
Hemodialysis (Y/N)	2/37	4/7	0.017
ECMO use (Y/N)	0/39	2/9	0.045
DCL wound open care (Y/N)	21/18	7/4	n.s.
Duration of laparotomy wound opened (days)	2.03 ± 2.91	1.11 ± 1.70	n.s
Accumulated blood Transfusion (U)	19.6 ± 4.16	32.9 ± 10.9	0.014

*SD*, Standard deviation; *APACHI II*, Acute physiology and chronic health evaluation II; *GCS*, Glasgow Coma Scale; *PaO2*, Arterial oxygen tension; *FiO2*, Fraction of inspiration oxygen; *WBC*, White cell count; *Hb*, Hemoglobin; *PLT*, Platelet; *INR*, International normalized ratio, for prothrombin time; *ECMO*, Extracorporeal membrane oxygenation; *DCL*, Damage control laparotomy.

### Multivariable analysis

Factors that were significant in abovementioned analyses were further enrolled for multivariable analysis. However, no significant variables were identified during further logistic regression analysis. Even when we enrolled only factors with *p* < 0.01, no factor remained statistically and independently significant.

## Discussion

DCL is a life-saving procedure. When this procedure is indicated, patients usually do not have any other choice for their treatment. The basic rationale of DCL is for hemorrhage and contamination control at the early, life-threatening period. After the DCL, the clinicians then return patients to relatively stable conditions, so the patients can undergo definitive surgical treatment at the next stage. Even with the development of new strategies to manage and resuscitate patients with severe trauma [8,9] and the lack of high level supporting evidence [10], DCL still plays an important role in trauma care, even though some clinicians have reflected on its futility [11,12].

Although DCL can bridge a patient with exsanguination from a devastating condition to a stage for definitive treatment, some patients still succumb to their critical condition even after successful hemostasis. In this study, we explored the factors that influenced patients’ outcomes after initially successful hemostasis. Our analysis included 3 different parts: demographic data and clinical conditions upon arrival at the ED, perioperative conditions, and early ICU parameters and intervention. In the univariable analysis, most of the significant factors were noted in the initial ED stage and the early ICU stage, while an analysis of perioperative factors revealed minimal survival impact. Initial hypoperfusion (pH, BE, and GCS level) and initial poor physiological conditions (body temperature, RTS, and CPCR at ED) may contribute to a patient’s final outcome. These factors are similar to the risk factors that were proposed by previous studies [13,14], while RTS itself has served as a surrogate for survival prediction [15,16].

The parameters recorded during the initial ICU admission represent the clinical conditions immediately after DCL. In addition to physiological and laboratory parameters, accumulated blood transfusion volumes, which was previously observed [17], and the use of inotropic agents also predict a dismal outcome. We also included use of organ support system in our analysis. Hemodialysis and ECMO applications are inevitable interventions for patients with life-threatening organ failure or temporary, irreversible organ function. In our study, all the studied subjects did not have predisposing organ failure. All conditions with organ failure and later hemodialysis or ECMO application were related to the deterioration of clinical course.

In our study, 11 subjects did not survive. We summarized the clinical profiles of these patients (Table 4). Almost half of these patients finally died due to brain death (4 patients due to initial brain injury, and 1 patient due to hypoxic encephalopathy). For these patients who died of brain death, 80% (4/5) died within the first week of admission (mean hospital stay, 6 days; median hospital stay, 4 days). For the other 6 patients, 5 of them died from infectious complication (4 from intra-abdominal origin, and 1 patient from low respiratory tract infection). Although a previous study identified low respiratory tract infection as the most common [18] type of post-DCL infection, intra-abdominal infection may contribute lethal effect to patients. Case #3 in Table 4 was a patient with Child A cirrhosis due to alcoholic hepatitis. He suffered from concurrent and relative low grade hepatic and splenic injury, which is why low ISS was noted. Although methods of laparotomy wound management and timing of abdominal closure after DCL influence the clinical outcome [19], these factors could not be well assessed in our series due to the small number of patients. In addition, patients who succumbed to infectious complications were typically older (Table 4). According to our study, late death for patients undergoing DCL may be attributed to an initial brain insult or an infectious complication, especially intra-abdominal infections.

**Table 4:** Summary of patients with mortality

	**Injury type**	**Age/gender**	**Initial GCS**	**RTS**	**CPCR at ED**	**ISS**	**APACHI II**	**OP times**	Accumulated transfusion*	**HD**	**ECMO**	**Cause and time of death (days)**
#1	Blunt	22/F	8	5.971	N	57	21	2	12	N	N	Brain stem failure (2)
#2	Penetrating	85/M	15	6.376	N	18	14	2	18	N	N	Sepsis with intra-abdominal infection (14)
#3	Blunt	60/M	15	4.918	N	4	31	3	68	Y	N	Hepatic failure (13)
#4	Blunt	18/M	3	3.361	N	45	22	2	44	N	N	Brain stem failure (6)
#5	Penetrating	50/M	10	6.904	N	18	15	3	16	Y	N	Sepsis due to pneumonia (31)
#6	Blunt	51/M	4	5.039	N	34	25	3	42	N	N	Sepsis with intra-abdominal infection (2)
#7	Blunt	19/M	3	1.95	Y	41	25	2	30	N	N	Brain stem failure (14)
#8	Blunt	25/M	6	5.097	Y	29	28	2	56	N	N	Brain stem failure (4)
#9	Blunt	23/M	3	0.872	Y	36	25	2	24	N	Y	Brian stem failure (4)
#10	Blunt	61/M	15	7.8412	N	30	24	2	32	Y	N	Sepsis due to ischemic bowel (3)
#11	Blunt	57/M	11	5.449	N	41	16	2	20	Y	Y	Sepsis due to intra-abdominal infection (25)

* Amount of total packed red blood cell and whole blood transfusion before ICU admission. *GCS*, Glasgow Coma Scale; *RTS*, Revised trauma score; *CPCR*, Cardiopulmonary cerebral resuscitation; *ISS*, Injury severity score; *APACHI II*, Acute physiology and chronic health evaluation II; *OP*, Operation; *HD*, Hemodialysis; *ECMO*, Extracorporeal membrane oxygenation.

Some previous studies proposed prediction factors or established prediction models for outcome prediction. However, most of these studies focused on overall clinical outcome [13,14,18,20]. No study has specifically emphasized the cause of death after hemostasis was achieved. These studies may be lacking due to the difficulty of performing these studies that assess DCL. Due to the improvement of non-operative treatment for abdominal trauma, especially for solid organ injury with internal hemorrhage, laparotomy is now not the only treatment option. This progress has made collecting suitable subjects difficult. In addition, heterogeneity has also been a big hurdle for analysis. Furthermore, a prospective study is likely impossible in this critical situation. Together, these unfavorable factors have contributed to the lack of high quality studies on this topic. In our study, we tried to eliminate the heterogeneity by enrolling only patients who were sent to the OR directly from the ED and who were injured within 6 hours of admission. In addition, we also eliminated patients who underwent DCL at another hospital and were then transferred to our hospital. However, we were still unable to obtain enough subjects for delicate statistical analyses, even when we attempted to use stringent rules by applying non-parametric analyses. Further, the multivariable analysis could not identify any independent risk factor because of the small size of the study sample. Finally, the studied subjects were observed over a 10-year period, and the impact of new medical and surgical progress may not be totally ignored.

## Conclusions

According to our study, the risk factors of late death for patients undergoing DCL may include both the initial status related to the trauma and the clinical conditions after DCL. In our series, the causes of death for patients with late mortality included an initial brain insult and later infectious complications. However, our study was unable to identify independent and statistically significant risk factors by multivariable analysis. The collection of more study subjects should be considered for future in depth analyses.

## Abbreviations

DCL: Damage control laparotomy; ICU: Intensive care unit; TAE: Trans-arterial embolization; CGNH: Chang Gung memorial hospital; ED: Emergency department; OR: Operating room; ISS: Injury severity score; RTS: Revised trauma score; GCS: Glasgow coma scale; CPCR: Cardiopulmonary and cerebral resuscitation; BE: Base excess; ECMO: Extracorporeal membrane oxygenation; APACHE II: Acute physiology and chronic health evaluation II; WBC: White cell count.

## Competing interests

Our co-authors report no personal conflicts of interest related to the study, and there was no funding from either the public or private sector related to the study.

## Authors’ contributions

All authors have made substantive contributions to the study: Study conception and design: L-ML, S-HW, and C-YF. Acquisition of data: C-HL, I-MK, S-CK, and S-WC. Analysis and interpretation of data: S-YW, C-HO, and Y-PH. Manuscript drafting: L-ML, S-HW, C-NY. Critical revision: S-HW and S-JY. All authors read and approved the final manuscript.
